# The quality and outcomes framework – QOF-transforming general practice

**Published:** 2012-12-18

**Authors:** Kirsten Hoeper

**Affiliations:** Institute for Epidemiology, Social Medicine and Health Systems Research, MedizinischeHochschule, Hannover, Carl-Neuberg-Straße 1, 30625 Hannover, Germany E-mail: hoeper.kirsten@mh-hannover.de

In the United Kingdom primary care is delivered through general practitioners (GP) and their work within the NHS is regulated by a national contract. Most GPs are private contractors, operating under this national contract and paid by Primary Care Trusts (PCTs). GPs are normally the first point of contact for patients and act as gatekeepers for access to secondary care services [1].

The Quality and Outcomes Framework (QOF) was introduced as part of the new GP contract in 2004 in order to meet the increasing pressure of health services to provide cost-effective high-quality care in times of an ever rising number of patients with various chronic conditions. The idea of linking physician’s pay to quality of care is to change the status quo by encouraging not only immediate but also long-term improvements in performance [2]. Practice participation in QOF is voluntary but most GPs participate in the pay-for-performance scheme [3]. GP practices score points according to their level of achievement based on indicators for how well the practice is organized, how patients view their experience, whether extra services are offered, and how well common chronic diseases are managed, and practice payments are calculated accordingly.

However, since its introduction, the effects of the QOF on quality of care have been the subject of constant and on-going discussion within the UK as well as internationally. The purpose of this book is to give an independent analysis of the impact of the QOF and to provide an insight into the evidence and critical reflection from varying perspectives.

The book is divided into four parts, with a total of 12 chapters:

Part I focuses on the background of the QOF, part II summarizes, reviews and analyses the research findings, part III provides insight on practical aspects, and part IV reflects on P4P within and outside the QOF. Each chapter starts with a summary and list of key points to be discussed.

The initial chapter of this book provides a short introduction to the topic, followed by an outline of the structure of the book including short summaries of each of the four parts.

Chapter 2 describes the development and implementation of the QOF from its early attempts in 1986 to its introduction in 2004 with a focus on the development of indicators and Chapter 3 provides insight into the further development of QOF after its introduction. The first part of the chapter describes one major (2006) and one minor (2009) reorganisation. The second part covers the new NICE-led process of developing QOF indicators.

Part II “Impact of the Quality and Outcomes Framework” includes chapters 4–7.

Chapter 4 reports on the published evidence about the effects of the QOF. Unfortunately, all studies were observational and a synthesis of data could not be conducted. Additionally, very little research about patients’ views was found. However, the authors found some weak evidence of cost-effectiveness and increasing equity.

Chapter 5 sets out to examine the potential impact of the QOF on Public Health, with a focus on health inequalities and area-based differences. The authors summarized that differences in performance between practices in deprived and non-deprived areas are narrowing. But evidence was reported to be limited of the direct impact of the QOF on health or health inequalities.

Chapter 6 critically reflects on informatics opportunities and challenges. On the one hand in-practice systems and electronic patient records provide researches with a huge database, but major limitations are described. Furthermore, the authors indicate that better recording results in higher QOF points, but at the same time may not necessarily be an indicator of better care. Finally, the QOF’s vulnerability to gaming and manipulation is examined.

Chapter 7 summarizes evidence from two sets of papers regarding the impact of QOF on practice organisation and service delivery. Findings of two linked qualitative case studies in England and Scotland in four very different general medical practices are reported. The authors show that even though all practices had changed their structures and organisation significantly, all four practices did not relate to the fact as a substantial change. Interestingly though, the observed changes narrowed the gap in difference among those practices in terms of structure, organisation and in type of care offered.

Part III with only two chapters focuses on practical aspects. Chapter 8 provides insight into ways for practices to maximise their QOF potential. Mixed with numerous practical examples the authors show how to achieve targets with forward planning and good organisation. Chapter 9 addresses the patient’s perspective. So far there has been little attention paid to patient related aspects of the QOF. Patients might find it difficult to distinguish between benefits for their own good or benefits in terms of financial rewards for the general practitioner.

Part IV with chapters 10–12 completes the book with a reflection on pay-for-performance (P4P) in primary care and an international perspective.

Chapter 10 looks into current P4P schemes and extent of contribution to quality improvement in primary care. The chapter starts by identifying key aspects of quality. However, defining quality is complex, and consequently various definitions are provided all over the literature. In the course of the chapter the authors present relevant literature and primary research, focussing on the effect of P4P on quality. The authors show that although evidence is limited, P4P schemes do have an effect on the behaviour of physicians, but broader definitions should be taken into account.

Chapter 11 provides an international perspective on P4P. The authors challenge the evidence base, flexibility of the system in terms of individual care, and cost-effectiveness, and discuss the importance of unintended consequences. They finish the chapter with pointing a view to alternative strategies of improving patient care.

In the final chapter the editors reflect on the contributions to the book with the question: “The Quality and Outcomes Framework: triumph of evidence or tragedy for personal care?” They point out the healthcare gains, the limitations, and the costs. The editors discuss changing roles and changing practice, what health professionals think and what patients want. They finalize the book with a critical focus on the limitations of the QOF and the need for more evidence.

Overall, this book can be recommended to all those interested in the future of general practice and primary care. The strength of the book is that it provides a very broad analysis of benefits and limitations, and sources of actual and potential conflicts of the QOF. Overall, modest improvements in the quality of care measured and slight reductions in disparities between socioeconomic groups are the main positive effects, but evidence on cost-effectiveness remains marginal. Pay-for-Performance schemes are part of a comprehensive integrated approach to improve the quality of care of patients with chronic conditions. It is hard to tell, if not impossible, to what extent benefits can be attributed to financial incentives. Nevertheless, the UK’s experience provides some lessons for other countries. A huge amount of money has been spent on implementing the QOF, yet, with limited evidence of improvement in health outcomes. Due to the comprehensive insight into the QOF and the very good structure, this book is of interest to readers directly involved in the QOF, but especially to those who have not previously worked on the subject and want to get a detailed overview, some empirical data and practical insights. Overall, a reader with a deeper interest in this field might be referred to the broad literature list.
